# Assessment of the Anticancer Effect of Chlorojanerin Isolated from *Centaurothamnus maximus* on A549 Lung Cancer Cells

**DOI:** 10.3390/molecules28073061

**Published:** 2023-03-29

**Authors:** Omar Noman, Fahd A. Nasr, Mohammad Z. Ahmed, Md Tabish Rehman, Wajhul Qamar, Ali S. Alqahtani, Sebastian Guenther

**Affiliations:** 1 Department of Pharmaceutical Biology, Institute of Pharmacy, University of Greifswald, 17489 Greifswald, Germany; 2 Department of Pharmacognosy, College of Pharmacy, King Saud University, Riyadh 11451, Saudi Arabia; 3 Department of Pharmacology and Toxicology, College of Pharmacy, King Saud University, Riyadh 11451, Saudi Arabia

**Keywords:** apoptosis, A549, cell cycle, *Centaurothamnus maximus*

## Abstract

The goal of this study was to assess the anticancer efficacy of chlorojanerin against various cancer cells. The effects of chlorojanerin on cell cytotoxicity, cell cycle arrest, and cell apoptosis were examined using MTT assay, propidium iodide staining, and FITC Annexin V assay. RT-PCR was employed to determine the expression levels of apoptosis-related genes. Furthermore, docking simulations were utilized to further elucidate the binding preferences of chlorojanerin with Bcl-2. According to MTT assay, chlorojanerin inhibited the proliferation of all tested cells in a dose-dependent manner with a promising effect against A549 lung cancer cells with an IC_50_ of 10 µM. Cell growth inhibition by chlorojanerin was linked with G2/M phase cell cycle arrest in A549 treated cells. Flow cytometry analysis indicated that the proliferation inhibition effect of chlorojanerin was associated with apoptosis induction in A549 cells. Remarkably, chlorojanerin altered the expression of many genes involved in apoptosis initiation. Moreover, we determined that chlorojanerin fit into the active site of Bcl-2 according to the molecular docking study. Collectively, our results demonstrate that chlorojanerin mediated an anticancer effect involving cell cycle arrest and apoptotic cell death and, therefore, could potentially serve as a therapeutic agent in lung cancer treatment.

## 1. Introduction

Cancer remains one of the largest global public health challenges despite the crucial progress made in cancer therapy using different treatment strategies [1]. The most popular method for treating cancer is still the use of conventional treatments, despite the fact that they are frequently associated with fatal side effects. However, natural sources are now considered as a promising source of new medications with fewer side effects. In the field of cancer research, plants are still a potential source for a novel chemical compound [2,3]. With a different biogeographic region, Saudi Arabia provides a remarkably rich source for medicinal plants that have anticancer properties [4,5].

*Centaurothamnus* genus is an Indigenous Centaurea monotypic species that inhabits the southwestern mountains of the Arabian Peninsula [6,7,8]. Only one species, *Centaurothamnus maximus*, belongs to the *Centaurothamnus* genus; it is endangered and grows in a very small geographic region on cliffs and steep hillsides [9]. *Centaurothamnus maximus* Wagentz and Dittri (Fam. Asteraceae) is a leafy shrub with many branches, which grows to about 1.5 m tall [7]. The leaves have a silvery undersurface and the magenta flowers are about 4 cm wide with a faint sweet scent. The plant is found in both Yemen and in the southern part of Saudi Arabia [10]. Sesquiterpene lactones, guaianolides, elemanolides as well as the germacranolides are considered the major phytochemical that are found in *C. maximus* species [11].

Chlorojanerin (a guaianolide-type sesquiterpene lactone) was previously isolated from several species of the *Centaurea* genus including *Centaurea hermannii* [12], *Centaurea musimomum* [13], *Centaurea solstitialis* [14], as well as *Centaurothamnus maximus* [10]. The biological studies of chlorojanerin have revealed that it possesses various effects such as anti-ulcerogenic [14], anti-inflammatory [15] and antiviral and antimicrobial activities [16]. Additionally, chlorojanerin has showed promising effectiveness in preventing the growth of various cancer cells [10,17]. However, there have been no previous reports regarding its molecular events associated with cell death. Herein, chlorojanerin was isolated from *C. maximus* and its antiproliferative effect was studied. In the following, we have demonstrated for the first time that chlorojanerin mediates cell cycle arrest and induces apoptosis in A549 lung cancer cells.

## 2. Results

### 2.1. Antiproliferative Effects of Chlorojanerin on Cancer Cell Lines

**Figure 1:**
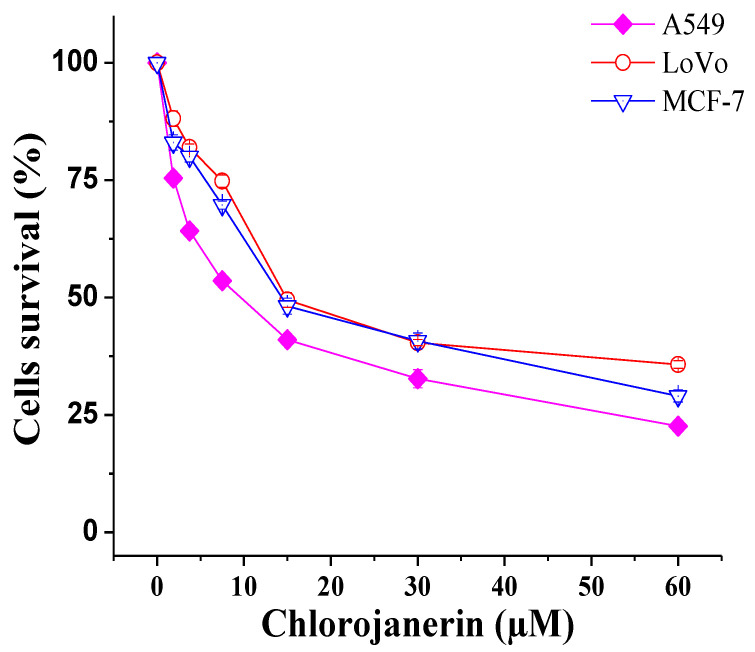
Cytotoxic effects of chlorojanerin on the viability of different human cancer cells measured by MTT assay. Cells were treated with various chlorojanerin concentrations (0–60 µM) for 24 h. Results are the mean ± S.D. from three separate experiments.

In the present study, we used MTT cell viability assay to evaluate the antiproliferative potential of chlorojanerin compound at different concentration. We determined that chlorojanerin exerted a remarkable reduction in the number of viable cells in a dose-dependent manner (Figure 1). Notably, A549 cells were more sensitive to chlorojanerin (IC_50_ = 10 µM) compared to other tested cells (Table 1). Based on these results, A549 cells and 5 µM and 10 µM concentrations of chlorojanerin were chosen for further assays.

### 2.2. Chlorojanerin Induces G2/M Cell Cycle Arrest in A549 Lung Cancer Cells

**Figure 2:**
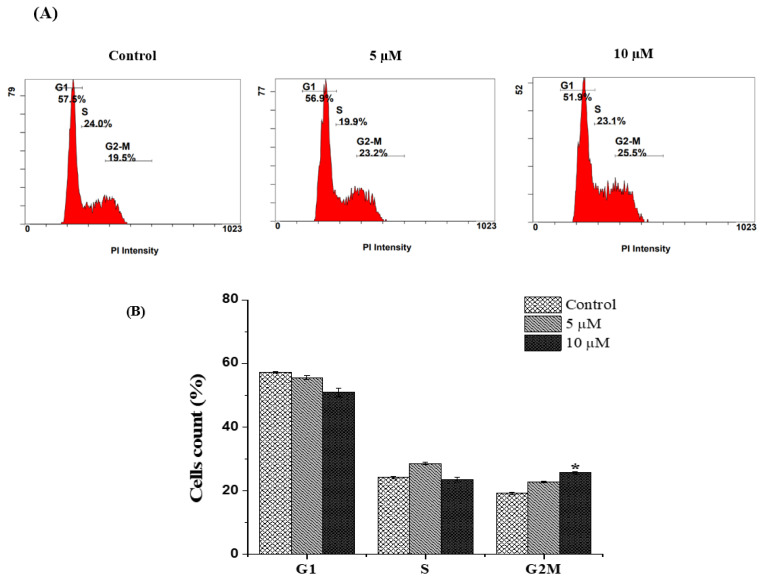
Effect of chlorojanerin on cell cycle phases of human lung cancer cells. A549 cells were treated with chlorojanerin (5 and 10 μM) for 24 h. (**A**) Representative flow cytometric histogram of cell cycle progression in the A549 after particular treatments. (**B**) Bar graph displaying the percentage of total population of cells. Results are presented as the mean ± S.D. from three experiments.; * *p* < 0.05 vs untreated control.

To clarify whether cell growth inhibition by chlorojanerin involves cell cycle changes, flow cytometry analysis was employed to examine the cell cycle phase distribution. A cell cycle analysis of chlorojanerin-treated A549 cells at 5 and 10 µM for 24 h revealed a remarkable concentration-dependent accumulation of A549 cells in the G2/M phase (increased to 22.85 ± 0.2% and 25.75 ± 0.35%, respectively) compared to the control (19.25 ± 0.35%). Simultaneously, chlorojanerin induced a notable reduction in cells in the G1 phase of the cell cycle (Figure 2). Our results suggested that chlorojanerin inhibited the cell cycle progression by inhibiting cells at the G2/M phase, which confirms the growth inhibition effect of chlorojanerin in A549 cells.

### 2.3. Chlorojanerin Promotes the Apoptosis of A549 Cells

**Figure 3:**
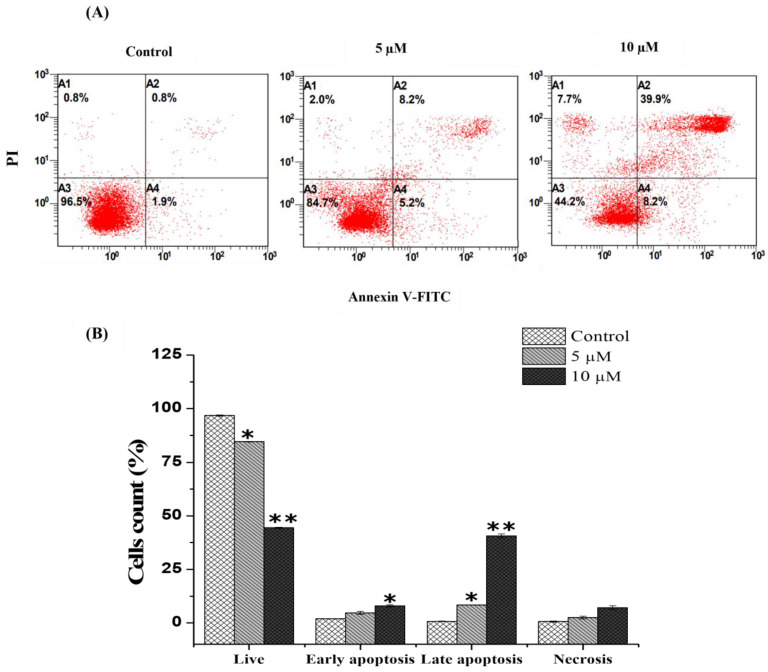
Effect of chlorojanerin on the apoptosis induction of A549 cells. A549 cells were treated with chlorojanerin for 24 h at 5 and 10 μM. (**A**) Annexin V-PI double staining histogram showing cell apoptosis. (**B**) Quantitative data of live, early and apoptotic cells and necrotic apoptotic cells. * *p* < 0.05; ** *p* < 0.01 vs untreated control.

Based on the cytotoxic action of chlorojanerin, the Annexin V FITC/PI Double Staining Kit was utilized to determine if chlorojanerin induced A549 cell apoptotic death mode. Flow cytometry analysis showed that the percentages of apoptotic A549 cells significantly increased with the increase in the concentration of chlorojanerin (Figure 3). Our results revealed that untreated control cells were viable (96.75 ± 0.35%), whereas A549 cells treated with chlorojanerin at the concentrations of 5 and 10 μM for 24 h exhibited a decreased percentage of viable cells to 84.6 ± 0.5 and 44. 6 ± 0.3, respectively. The percentage of early apoptotic cells increased to 4.7 ± 0.7% and 7.9 ± 0.4%, respectively, while a significant increment of late apoptotic cells was detected, with an increase to 8.25 ± 0.1% and 40.6 ± 0.9%, respectively. These findings showed that chlorojanerin treatment considerably increased the percentage of late apoptotic cells compared to the control (Figure 3).

### 2.4. Chlorojanerin Modulated the Expression of Apoptosis Related Genes

**Figure 4:**
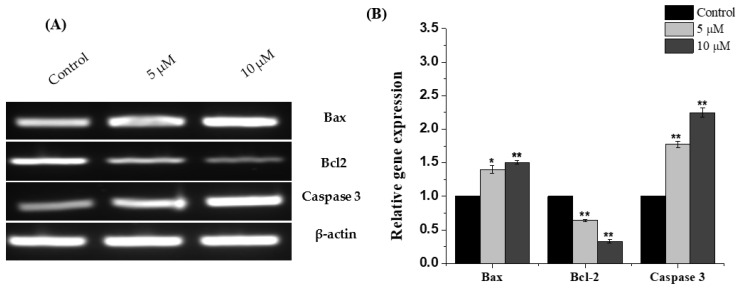
Effects of chlorojanerin on the mRNA expression of Bax, Bcl-2 and caspase-3 in human lung A549 carcinoma cells. (**A**,**B**) Fold-ratio: relative gene expression to control (control fold ratio: 1). Mean values are significantly different from control * *p* < 0.05 and ** *p* < 0.01.

To further understand the effectiveness of chlorojanerin in A549 cell death mode, the levels of mRNA expression for apoptosis-related genes was explored. By means of RT-PCR, it was established that, in comparison to untreated cells, the mRNA of Bax and caspase-3 were upregulated in A549 cells after chlorojanerin treatment (Figure 4). Moreover, our results showed that Bcl-2 was downregulated in chlorojanerin-treated A549 cells in a dose-dependent manner (Figure 4). 

### 2.5. Chlorojanerin Modulated the Expression of Apoptosis Related Proteins

**Figure 5:**
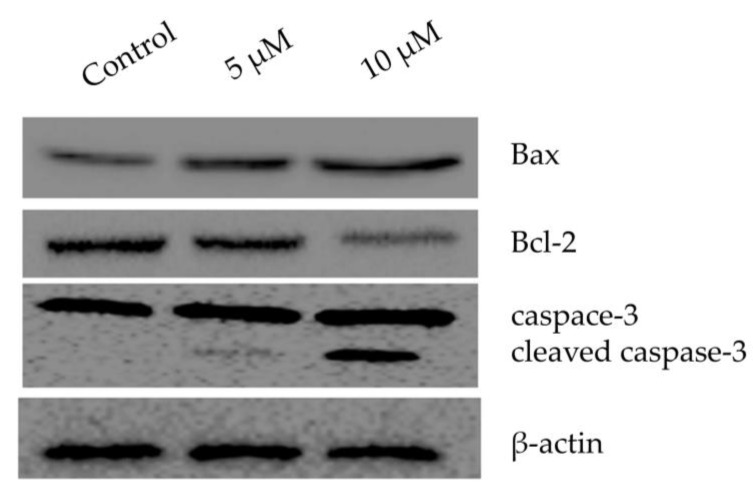
Effects of chlorojanerin on the protein expression of Bax, Bcl-2 and caspase-3 in human lung A549 carcinoma cells. Cells were treated with or without increasing concentrations of chlorojanerin for 24 h. Western blot analysis was carried out using specific antibody for each target gene while β-actin was served as an internal control.

Using Western blot analysis, the expression of proteins linked to apoptosis was assessed. As displayed in (Figure 5), the Bax expression was increased along with the increase in dose of chlorojanerin in comparison to the control. Chlorojanerin treatment also cleaved the caspase-3 in the treated cells, while the Bcl-2 expression was downregulated.

### 2.6. Molecular Docking

**Figure 6:**
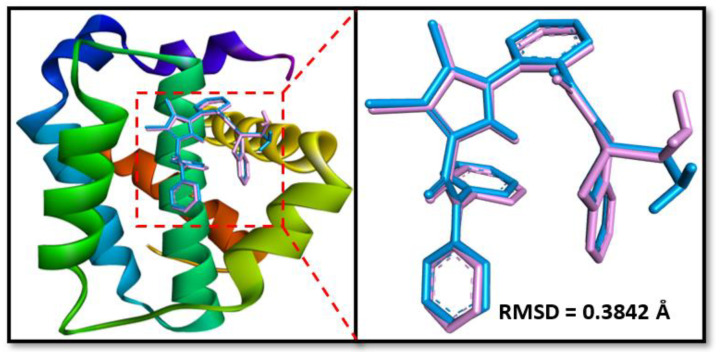
Validation of the docking protocol by extracting and re-docking the ligand (originally bound to the protein in the X-ray crystal structure). RMSD was calculated by superimposing the docked and crystal structure poses of the ligand. Chemically DRO is 1-(2-{[(3*S*)-3-(aminomethyl)-3,4-dihydroisoquinolin-2(1*H*)-yl]carbonyl}phenyl)-4-chloro-5-methyl-*N*,*N*-diphenyl-1*H*-pyrazole-3-carboxamide.

Prior to performing molecular docking between protein and ligand, validation of the molecular docking protocol was performed as described earlier [18]. The X-ray-bound ligand, namely DRO, was removed from the X-ray crystal structure and re-docked to target protein Bcl-2. The X-ray bound pose of the ligand was compared with the re-docked pose, and the root mean square deviation (RMSD) was calculated. The RMSD between the re-docked and crystal structure poses of DRO was estimated as 0.3842 Å (Figure 6). We determined that the RMSD (0.3842 Å) between the two poses was within the acceptable limit (2.0 Å), confirming a valid docking protocol to envisage the binding of chlorojanerin with Bcl-2.

**Figure 7:**
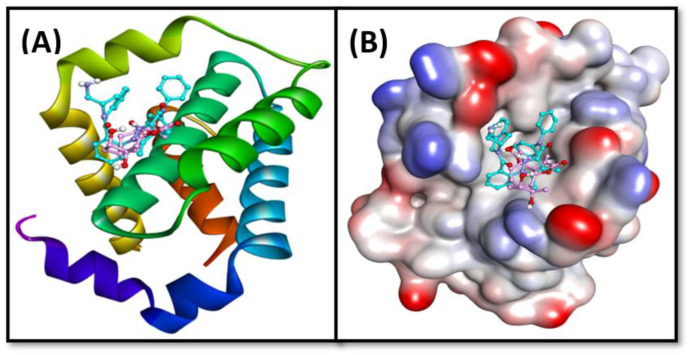
Binding of DRO and chlorojanerin at the active side of Bcl-2. (**A**) 2D cartoon representation, and (**B**) 3D representation of the active site and bound ligands.

**Figure 8:**
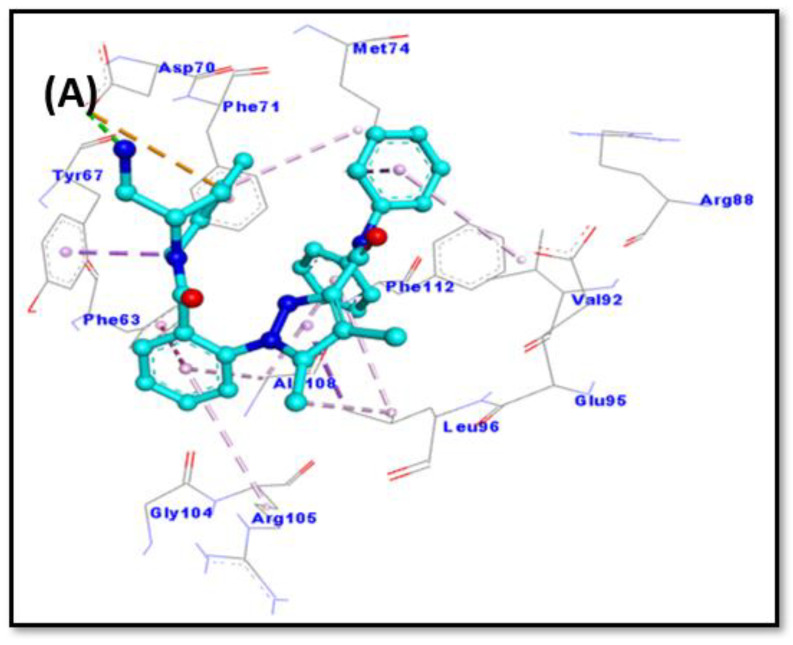

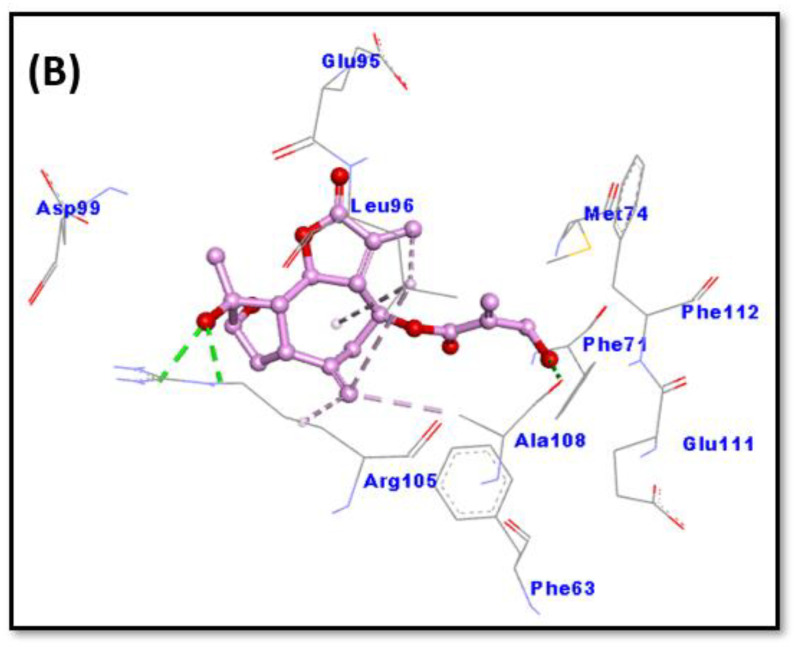
Molecular interaction between Bcl-2 and selected ligands. (**A**) Bcl-2 and DRO interaction, and (**B**) Bcl-2 and chlorojanerin interaction.

The analysis of molecular docking results suggests that chlorojanerin and DRO occupied a common position at the active site of Bcl-2 (Table 2, Figure 7). In DRO-Bcl-2 complex, a hydrogen bond between N-atom of DRO and OD2-atom of ASP70 along with an electrostatic interaction between DRO and ASP70:OD2 residue of Bcl-2 were the driving forces that stabilize the whole complex (Figure 8A). DRO also formed three π-σ hydrophobic interactions with MET74: CE, LEU96:CD1, and TYR67. In addition, DRO interacted with Bcl-2 through two π-π T-shaped hydrophobic interactions (with PHE63 and PHE71), and one Alkyl hydrophobic interaction with LEU96. Moreover, ARG105, ALA108, MET74, and VAL92 were engaged in forming π-alkyl hydrophobic interactions with Bcl-2 (Table 2). Further, some residues of Bcl-2 such as ARG88, GLU95, GLY104, and PHE112 were involved in van der Waals’ interaction, which in turn provide strength to the Bcl-2 and DRO complex. Similarly, an in-depth analysis of chlorojanerin and Bcl-2 complex formation indicates that there were three hydrogen bonds with Arg105: HE, ARG105:HH21, and ALA108:O responsible for the formation of a stable protein–ligand complex (Figure 8B). In addition, it also formed four Alkyl hydrophobic interactions (with LEU96, ARG105, and ALA108), and one π-alkyl hydrophobic interaction with LEU96 (Table 2). In addition, chlorojanerin formed van der Waals’ interactions with PHE63, PHE71, MET74, GLU95, ASP99, GLU111, and PHE112 to further strengthen the protein–ligand interaction (Table 2 and Figure 8B). Comparatively, the binding energies of DRO and chlorojanerin towards Bcl-2 were estimated to be −10.2 kcal mol^−1^ corresponding to binding affinity of 3.03 × 10^7^ M^−1^, and −6.5 kcal mol^−1^ corresponding to binding affinity of 5.85 × 10^4^ M^−1^, respectively (Table 1). Interestingly, PHE63, PHE71, MET74, GLU95, LEU96, ARG105, ALA108 and PHE112 residues of Bcl-2 were commonly involved in the interaction with both chlorojanerin and DRO (control ligand).

## 3. Discussion

Cancer remains one of the leading causes of disease-related death worldwide. Numerous traditional plant-derived compounds have demonstrated efficacy and safety in cancer treatment. Among natural plant products, sesquiterpene lactones still represent an interesting source of therapeutic bioactive compounds that exhibit cytotoxic properties [19,20]. In our exploration of anticancer agents from *C. maximus,* we have previously reported that janerin, a sesquiterpene lactone, exhibited growth inhibitory effects against leukemia cancer cells [21]. In the current study, we isolated another sesquiterpene lactone from *C. maximus* and assessed its cytotoxic potential and the underlying mechanism. Chlorojanerin (a guaianolide-type sesquiterpene lactone) was previously shown to inhibit different cancer cells [10]. In this study, we determined that the greatest cytotoxic effects of chlorojanerin occurred in A549 cells. Based on the IC50 values obtained from an MTT assay, A549 cells were selected to further investigate the impact of chlorojanerin on the cell cycle progression and apoptosis induction in A549 cancer cells. Consistent with the cytotoxicity results reported here, Tastan et al. reported that chlorojanerin isolated from *Psephellus pyrrhoblepharus* inhibited HeLa cell proliferation at 10 μM concentration [17], which was close to the results obtained in this study.

It is well known that uncontrolled cell division is associated with cancer progression and cell cycle arrest is considered a crucial regulatory mechanism to control cell division. Thus, targeting the cell cycle in cancer cells is considered a promising strategy for the management of malignant diseases [22]. In particular, the G2/M checkpoint is considered a crucial cell cycle checkpoint in the treatment of cancer because it enables cells with damaged DNA to repair the damage [23]. In this study, we show that chlorojanerin caused an accumulation of cells in the G2/M phase after 24 h of treatment. The scenario described here for the induction of G2/M phase cell cycle arrest in suppressing the proliferation of A549 cells has been demonstrated in our previous work with janerin compound that induced G2/M phase cell cycle arrest in human leukemia cancer cells [21].

Apoptosis is a controlled cell death process that is essential for the internal integrity and growth of both healthy and malignant cells. Dysregulation of apoptosis is frequently identified as a key characteristic of cancer [24]. In this study, annexin V-FITC and PI double staining, which can distinguish between living, apoptotic and necrotic cells [25], were used to label A549 cells treated with chlorojanerin. Our annexin V/PI staining experiment demonstrated that apoptosis is the primary mechanism involved in chlorojanerin-mediated cell death. In our previous study [21], the THP-1 leukemic cell line also showed similar apoptotic patterns following janerin treatment.

The family of Bcl-2 genes is crucial for cellular apoptosis regulation since some of its members, such as Bax, can trigger apoptosis, while others, such as Bcl-2, can halt it. Hence, there has been more focus on searching for anticancer drugs targeting these genes [26]. Consequently, the expression levels of these markers were assessed after chlorojanerin treatments. The upregulation of pro-apoptotic (Bax) and downregulation of anti-apoptotic gene (Bcl-2) detected in this study further confirm the occurrence of apoptotic events in A549 lung cancer cells.

The activation of caspases is also the crucial link in the apoptosis mechanism. In particular, caspase-3 activation led to amplification and initiation of apoptotic cell death [27]. Our results showed that caspase-3 expression was enhanced in A549 cells exposed to chlorojanerin compared to control (untreated) cells. These findings collectively offer more evidence for the apoptotic effects of chlorojanerin in lung cancer cells.

Chlorojanerin molecular docking simulations with Bcl-2 protein were also performed to better clarify its cytotoxic action. In fact, a variety of chemical compounds were identified and designed based on computer modeling (in silico approach) to study the interactions among Bcl-2 family proteins [28]. The comprehensive simulation results of chlorojanerin with the anti-apoptotic Bcl-2 protein (Table 2) showed a vital role in predicting molecular interactions of chlorojanerin with the targeted protein. The targeted protein in this study was Bcl-2 which is a well-known anti-apoptotic protein that participates in the regulation of apoptotic cell death [29].

## 4. Materials and Methods

### 4.1. Plant Collection, Extraction and Fractionation

In March 2019, the *C. maximus* aerial parts were gathered from Al-Baha province, Saudi Arabia. The plant species was verified by Prof. Mothana at the Pharmacognosy Department. A dry powder (500 g) from the aerial parts was extracted with ethanol using a Soxhlet device (3 L). Next, the liquid extract was dried up using a rotary evaporator and about 57 g of ethanolic extract was obtained. The latter was gradually partitioned to various polar solvents, including n-hexane, chloroform, and n-butanol, which contained 3.5, 17, and 23 g of dried fractions, respectively.

### 4.2. Compound Isolation and Identification

Using silica gel column chromatography, *Centaurothamnus maximus* compound 2 (CMA-2) (20 mg) was separated as previously described [21]. Briefly, 5 g of the chloroform fraction was applied to the column that packed with a silica gel (72 g, 80 cm × 3 cm). Elution was started with 3% of methanol: chloroform solvents. Gradient elution was increased with methanol that yielded 13 fractions (20 mL each). The matching fractions were gathered and grouped together based on TLC behavior which yielded seven major fractions. The antiproliferative activity was recorded in fraction D (47 mg), hence fraction D was further subjected to elution with 30% chloroform: methanol, which then re-chromatographed on a silica gel column (7.2 g, 60 cm × 1 cm) resulting in compound CMA-2 (20 mg). For the purpose of determining the chemical structure, a number of spectroscopic methods, including 1D and 2D-NMR, were utilized.

**Figure 9:**
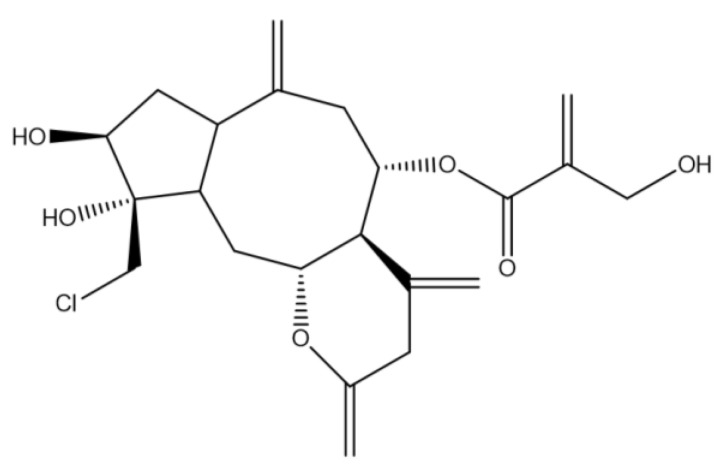
Chemical structure of chlorojanerin, which was isolated from the chloroform fraction of *C. maximus*.

Chlorojanerin (Figure 9), was isolated as white needles. Its mass spectral data suggested a molecular formula of C_19_H_23_ClO_7_. ^1^H NMR (700 MHz, DMSO-*d_6_*) δ 6.17 (s, Ha-18), 5.99 (s, Ha-13), 5.90 (s, Hb-18), 5.49 (s, Hb-13), 5.17 (s, Ha-14), 4.99 (m, H-8), 4.85 (s, Hb-14), 4.63 (t, *J* = 9.9 Hz, H-6), 4.17 (s, H-19), 3.22 (m, H-7), 3.10 (m, H-1), 3.06 (s, Ha-15), 2.96 (s, Hb-15), 2.69 (d, *J* = 14.3 Hz, Ha-9), 2.27 (d, *J* = 14.6 Hz, Hb-9), 2.21 (m, H-2). ^1^H NMR and ^13^C NMR was also reported by Saklani et al. [15].

### 4.3. Cell Culture Conditions

Human lung adenocarcinoma (A549), human breast adenocarcinoma (MCF-7) and colon adenocarcinoma (LoVo) cancer cell lines were obtained from the German Collection of Microorganisms and Cell Cultures (DSMZ) (Braunschweig, Germany). Cells were grown in DMEM medium (Gibco, part of Thermo Scientific, Waltham, MA, USA), supplemented with 10% heat-inactivated fetal bovine serum, FBS (Gibco), 1% penicillin/streptomycin under standard cell conditions in 5% of CO_2_ in an incubator at 37 °C.

### 4.4. Cell Viability Assay

The cytotoxicity of chlorojanerin against cells was determined using MTT assay. In brief, cells were plated in 96-well plates at a density of 5 × 10^4^ cells/well and incubated overnight at 37 °C. Various concentrations of chlorojanerin were then added (from 0 to 60 μM). Doxorubicin (a widely used as a chemotherapeutic agent for the treatment for various cancers) was used as a positive control, whereas DMSO (0.1%) was used as a vehicle. After 24 h, 10 μL of MTT (5 mg/mL) was added to each well and incubated for 4 h at 37 °C. The reduced MTT was measured at a wavelength of 570 nm using a microplate reader (BioTek, Winooski, VT, USA). Cell viability was calculated and the corresponding IC_50_ (concentration that produces 50% inhibitory effect on cell growth) was determined using OrignPro 8.5 software (Originlab Corporation, Northampton, MA, USA). The cell survival percentage was calculated as follows: Cell Viability (%) = (O.D of treated sample)/(O.D of untreated sample) × 100%

### 4.5. Cell Cycle Analysis

The chlorojanerin impact on cell cycle distribution was determined following previously described protocols [28]. Cells were seeded in 6-well plates overnight and then exposed to chlorojanerin at 5 and 10 μM concentration. After 24 h of exposure, cells were collected, washed with PBS, and fixed in cold 70% ethanol. The cells were pelleted again and resuspended in propidium Iodide (PI) + RNase staining solution followed by incubation at 37 °C in the dark for 30 min. Flow cytometer used: Cytomics FC 500 (Beckman Coulter, Brea, CA, USA). Data analysis was performed using CXP software 3.0 (Beckman Coulter, CA, USA).

### 4.6. Detection of Apoptosis by Flow Cytometry (Annexin V-FITC/PI Staining)

The A549 cells were exposed to chlorojanerin 5 and 10 μM concentrations for 24 h, and cell apoptosis was investigated using the FITC Annexin V apoptosis detection kit (Biolegend, San Diego, CA, USA). After the treatment of the cells, they were harvested, washed with PBS and resuspended in a binding buffer (100 µL). Thereafter, 5 µL of each Annexin FITC and PI dyes was added and further incubated for 15 min in the dark. Subsequently, 400 µL of annexin binding buffer was added to each tube and the cells were directly analyzed using a FACS Scan Flow Cytometer (Cytomics FC 500; Beckman Coulter, Brea, CA, USA). CXP software 3.0 was employed to analyze the obtained data.

### 4.7. Reverse Transcription Polymerase Chain Reaction (RT-PCR)

After chlorojanerin treatment (5 and 10 μM), total RNA was isolated from treated and untreated A549 cells using Trizol reagent (Invitrogen, USA) according to the manufacturer’s instructions. Reverse transcription was performed using total RNA with the Super Script VILO cDNA Synthesis Kit (Invitrogen, Waltham, MA, USA) using an equal amount of RNA (1 µg) in a final volume of 20 µL. The cDNA produced was utilized to assess the mRNA expression of apoptotic genes (Bax, Bcl-2 and caspase-3) using a specific primer for each gene while the β-gene was employed as a reference gene. The amplification RT-PCR products were electrophoresed on a 1.2% agarose gel containing ethidium bromide, and the gel was photographed using a Licor device (Licor, NE, USA).

### 4.8. Western Blot Analysis

As described before [21], a Western blot analysis was carried out to explore the impact of chlorojanerin on the apoptosis linked proteins. In brief, after exposure to chlorojanerin at 5 and 10 μM concentrations, proteins were extracted out from the treated and untreated A549 cells by using RIPA lysis buffer (Thermo Scientific, Waltham, MA, USA). The lysates were centrifuged at 13,000× *g* for 15 min at 4 °C and the supernatant were collected in a different Eppendorf tube. Equal concentration of protein was loaded on 12% SDS-PAGE, which was further followed by transferring of protein onto polyvinylidene disulfide (PVDF) membrane (Bio-Rad, Hercules, CA, USA). The membrane was incubated with the primary antibody (Bax, Bcl-2, caspase-3 and β-actin) for an overnight period at 4 °C after blocking with 5% BSA (Bovine serum Albumin) for an hour at room temperature. Binding of an HRP-conjugated secondary antibody was carried out the next day for an hour at room temperature. Using ECL reagent (GE Health care), the membranes were made visible, and pictures were taken using a Bio-Rad gel imaging device. To confirm equal loading across lanes, the membrane was stained for β-actin.

### 4.9. Molecular Docking Method

In order to evaluate the binding site and the nature of interactions involved, docking between chlorojanerin and Bcl-2 was executed as published previously [18]. The 3D coordinates of the target protein, i.e., Bcl-2, were downloaded from the X-ray crystal structure (Pdb Id: 2w3i; resolution was 2.10 Å) in which DRO was bound to Bcl-2 [30]. Prior to docking, protein molecule was treated to remove non-essential solvent molecules and any other foreign atoms. Further, new polar hydrogen atoms were supplemented while bond orders were re-defined, and rotatable bonds were again assigned [31]. A network of hydrogen bonds was redefined, and an MMFF force field (Merck Molecular Force Field) was used to minimize the energy of the protein. A grid box of 27 × 30 × 26 Å dimensions was placed at 39 × 28 × −12 Å with 0.375 Å spacing between the grid points. Finally, docking between protein and ligand was executed using Autodock 4.2 (Scripps Research, San Diego, CA, USA) [32]. Molecular docking was performed employing Lamarckian Genetic Algorithm (LGA) global search method and Solis and Wets local search methods. Initial position, orientation and torsions of ligand were set arbitrarily. Maximum 2.5 × 10^6^ calculations were performed for each docking run. Other parameters were set to their default values. The results were analyzed using Discovery Studio Visualizer (BIOVIA, San Diego, CA, USA). The binding affinity (*K_d_*) of chlorojanerin towards Bcl-2 was determined from binding energy (Δ*G*) using the following relation [33]:

$$\Delta G=-RTlnK_{d}$$

where *R* and *T* are the universal gas constant (1.987 kcal mol^−1^ K^−1^) and temperature (298 K), respectively.

### 4.10. Statistical Analysis

The data are displayed as mean standard deviation (S.D.) of three replicates. Statistical comparison was performed using the Student’s t-test. A significant difference was identified (*p* < 0.05).

## 5. Conclusions

In conclusion, this study has shown that chlorojanerin inhibits cancer cell proliferation in vitro. The cell viability assay showed that chlorojanerin was most potent against A549 lung cancer cells. Inhibition of A549 cell proliferation was associated with G2/M phase cell cycle arrest and apoptosis induction. Our findings imply that chlorojanerin may represent a novel therapeutic agent for lung cancer management. These findings provide new insight into the effects of chlorojanerin. However, there is a need for further research to confirm the detailed mechanism of chlorojanerin in vitro, as well as an in vivo animal model to verify its impact and provide additional data regarding its underlying mechanism.

## Figures and Tables

**Table 1:** IC_50_ values of chlorojanerin and standard doxorubicin for different cancer cells.

Compound	Cells and IC_50_ (µM)		
	A549	LoVo	MCF-7
Chlorojanerin	10.0 ± 0.4	15.9 ± 0.8	15.5 ± 0.7
Doxorubicin	1.58 ± 0.02	2.35 ± 0.05	1.74 ± 0.07

**Table 2:** Molecular docking of chlorojanerin and DRO (control ligand) with Bcl-2.

Donor Atoms	Acceptor Atom	Distance (Å)	Type of Interaction	Binding Free Energy, ΔG (kcal mol^−1^)	Binding Affinity, *K*_d_ (M^−1^)
Bcl-2 and DRO * complex					
LIG:N ASP70:OD2 MET74:CE LEU96:CD1 LIG:C PHE63 PHE71 LIG:C LIG LIG LIG LIG LIG LIG	ASP70:OD2 LIG LIG LIG TYR67 LIG LIG LEU96 ARG105 ALA108 MET74 LEU96 ALA108 VAL92	2.9553 4.8135 3.5674 3.8681 3.5199 5.2880 4.9682 4.9539 5.2941 4.4414 5.4984 5.1674 4.4935 5.0873	Conventional Hydrogen Bond Electrostatic (π-Anion) Hydrophobic (π-σ) Hydrophobic (π-σ) Hydrophobic (π-σ) Hydrophobic (π-π T-shaped) Hydrophobic (π-π T-shaped) Hydrophobic (Alkyl) Hydrophobic (π-Alkyl) Hydrophobic (π-Alkyl) Hydrophobic (π-Alkyl) Hydrophobic (π-Alkyl) Hydrophobic (π-Alkyl) Hydrophobic (π-Alkyl)	−10.2	3.03 × 10^7^
Bcl-2 and chlorojanerin complex					
ARG105:HE ARG105:HH21 LIG:O LEU96 ALA108 LIG:C LIG:C LIG:C	LIG:O LIG:O ALA108:O LIG LIG:C LEU96 ARG105 LEU96	2.5827 2.5827 2.7004 5.2020 3.6803 5.0962 4.1620 4.4392	Conventional Hydrogen Bond Conventional Hydrogen Bond Conventional Hydrogen Bond Hydrophobic (Alkyl) Hydrophobic (Alkyl) Hydrophobic (Alkyl) Hydrophobic (Alkyl) Hydrophobic (π-Alkyl)	−6.5	5.85 × 10^4^

* Chemically DRO is 1-(2-{[(3*S*)-3-(aminomethyl)-3,4-dihydroisoquinolin-2(1*H*)-yl]carbonyl}phenyl) -4-chloro-5-methyl-*N*,*N*-diphenyl-1*H*-pyrazole-3-carboxamide.

## Data Availability

The data presented in this study are available on request from the corresponding author.

## Sample Availability

Samples are available from the authors upon reasonable request.
